# Acute Paraplegia in a Toddler: A Diagnostic Journey Compounding the Challenge in Management: A Case Report

**DOI:** 10.5704/MOJ.1607.012

**Published:** 2016-07

**Authors:** JH Goh, M Fazir, NA Zainal-Abidin, D Amir, M Singh

**Affiliations:** Hospital Kuala Lumpur, Kuala Lumpur, Malaysia

**Keywords:** Atypical spinal tuberculosis, paediatric, extradural tuberculoma, paraplegia

## Abstract

Spinal tuberculosis is not common in the paediatric age group. Initial clinical features are often vague and non specific until the disease progresses to later stages. We highlight the diagnostic difficulties and management challenges of a complicated extradural tuberculoma with neurological deficits in a very young girl.

## Introduction

Atypical spinal tuberculosis, occurring in an uncommon age group is very difficult to diagnose. Diagnosis is usually delayed and ascertained only after a barrage of investigations before initiation of treatment^1^. With the rapidly increasing emergence of multidrug resistance tuberculosis, instituting chemotherapy must be done carefully. Surgical procedures remain an adjunct to chemotherapy.

## Case Report

A 2 years 6 months old girl presented with a week’s history of paraplegia and defecation difficulties. The history started two months prior to referral to our hospital with a trivial fall at home on the stairs, in which she managed to break her fall with her hands. There was no trauma to her head, chest, back or pelvis. There was no immediate complaint of pain nor neurological deficit and she was well thereafter.

Two days after the fall, she developed low grade fever, persistent back and abdominal pain aggravated by movement. She was taken to a general practitioner who had found no abnormality in radiographs of the spine and pelvis. A diagnosis of acute gastroenteritis had been made and she was prescribed antibiotics and analgesic. Her symptoms worsened and she was taken to a district hospital, where the doctors confirmed no abnormalities in her spinal and pelvic radiographs and also found urine microscopy examination normal. The diagnosis of gastroenteritis was retained and symptomatic medication prescribed.

A week later, she had difficulty passing motion, in addition to her low grade fever and back pain. The mother took her to another general practitioner and was diagnosed as constipation and treated with laxatives.

Two weeks later, her lower limb weakness worsened and she was no longer able to walk. She was then taken to a general hospital and subsequently referred to our center for further management.

She had no loss of weight nor any significant past medical or surgical history. She was the youngest child with four older healthy siblings. She was being taken care of by her father at home and there was no family history of tuberculosis.

Clinical examination revealed an alert and generally healthy child. She was comfortable with no syndromic facies, skin blemishes or birth marks. She had fever of 37.7 degrees Celsius. Vital signs were normal. Pupils were 3mm bilaterally and reactive with no photophobia. Examination of her back was normal with no step deformity, bruises, gibbus, cutaneous lesion nor paravertebral muscle spasm.

Neurological examination revealed motor power MRC grade 0 from L2 myotome down. Her lower limbs were hypertonic, hyper-reflexic, with clonus and up going Babinski reflex. Sensory evaluation revealed numbness below the level of her xyphisternum.

**Fig. 1:**
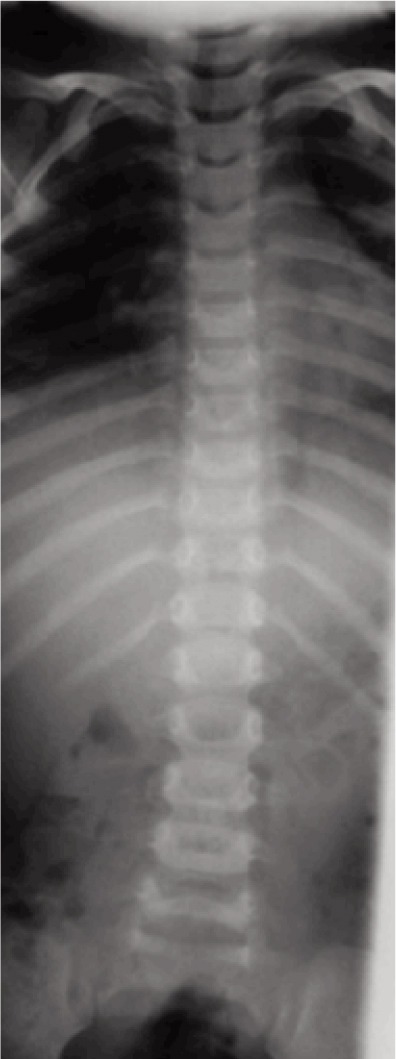
Pre-op AP radiograph.

Blood investigations were within normal range, ESR was 30mm/hr. Thoracic spine radiograph revealed reduced T10/T11 disc height and end plate erosion (Figure 1, 2a, 2b). CT brain scan with contrast was normal.

**Fig. 2a:**
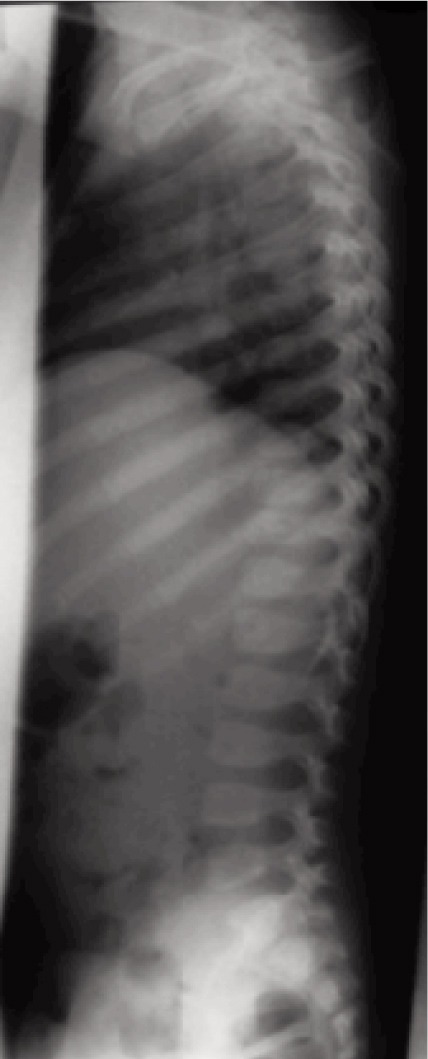
Pre-op lateral radiograph.

**Fig. 2b:**
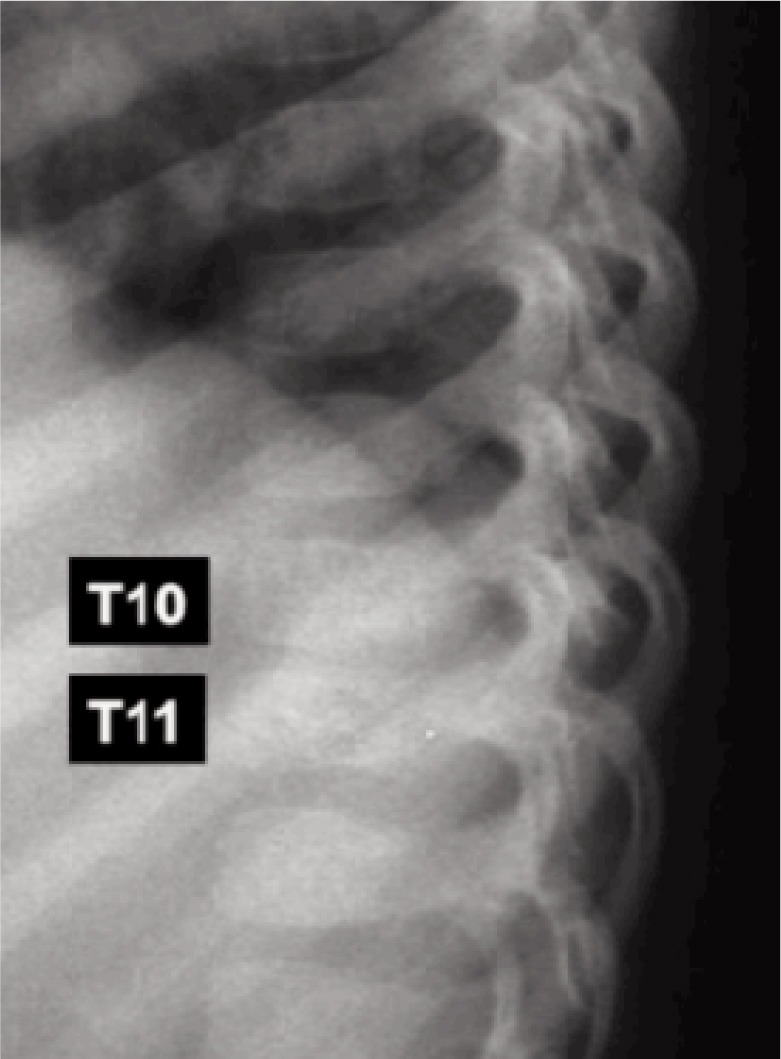
Enlarged lateral radiograph showing reduced T10-T11 disc height.

The key features of her MRI were partially collapsed T10 and T11 body with posterior cortex destruction and bulging posterior longitudinal ligament.

Marrow signal within T10 and T11 was hypointense in T1, hyperintense in T2 weighted sequence and post-gadolinium heterogenecity. There was no blooming artifact on gradient recalled echo (GRE) images. Alignment was intact.

Posterior half of her T10/11 disc was destroyed and end plates eroded. There was presence of thick walled paravertebral soft tissue collection with epidural extension from T9-T12 with cord compression and oedema (Figure 3a, 3b, 3c).

**Fig. 3a:**
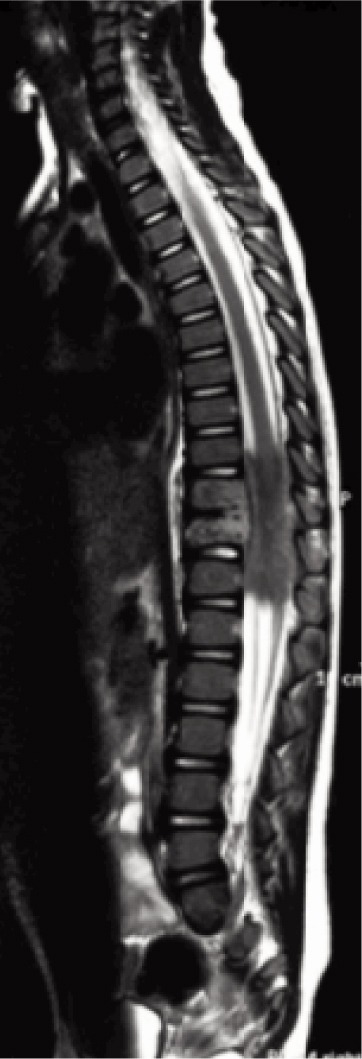
T2 weighted whole spine MRI.

**Fig. 3b:**
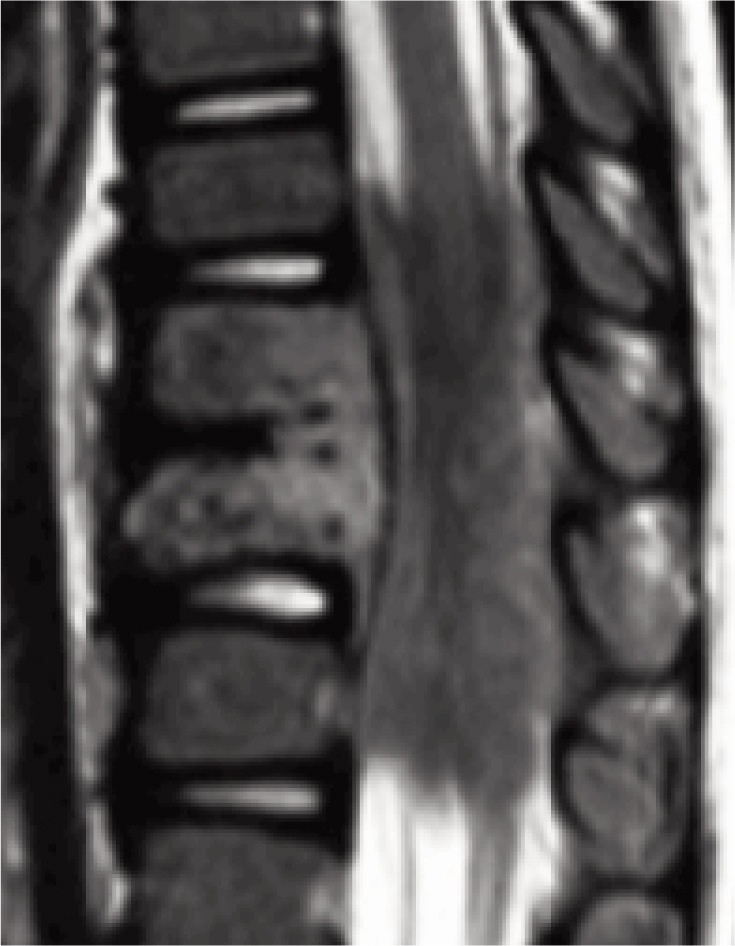
Enlarged T2 weighted MRI from T9 to T12.

**Fig. 3c:**
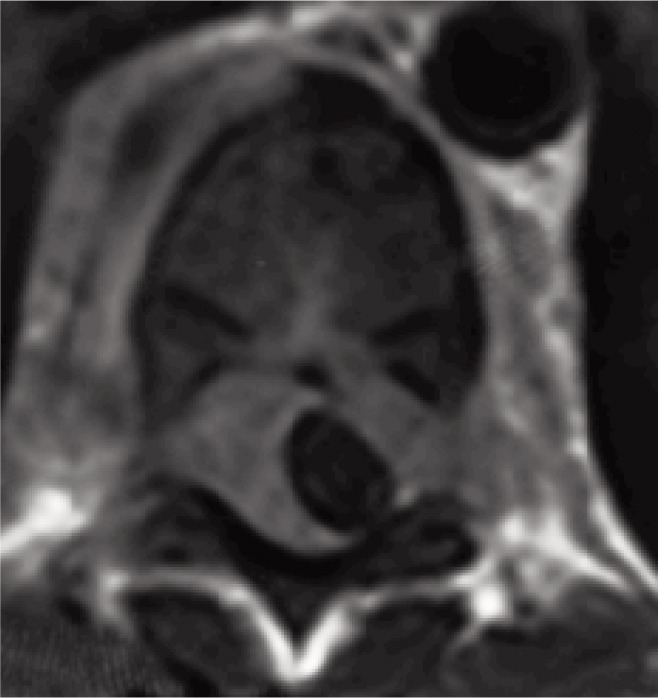
MRI axial view of T10.

The MRI features were suggestive of an infective process with differential diagnosis of tuberculosis, Langerhans cell histiocytosis, leukaemia, lymphoma and metastatic tumour.

Mantoux test was positive at 12mm.

After analysing her clinical features and investigations, we came to the working diagnosis of spinal tuberculosis with thoracic myelopathy with the differential diagnosis of haematological malignancies.

Posterior decompression surgery was decided as there was no significant anterior column destruction and instability. Emergency laminectomy at T10 and T11 levels was carried out, which however was technically challenging.

Special efforts were made to preserve the facet joints to avoid post-operative instability. There was no frank pus nor slough present. A thick walled whitish soft tissue of firm consistency was identified (Figure 4a). This tissue was found encasing the spinal cord. Gentle and careful dissection separating the soft tissue off the spinal cord was done, with a neurosurgeon on standby. The risk of dural tear was high with the presence of infection and tissue adhesions. Manipulation of her cord to allow clearance of the infective cord encasing tissue also posed risks of neurological complications.

**Fig. 4a:**
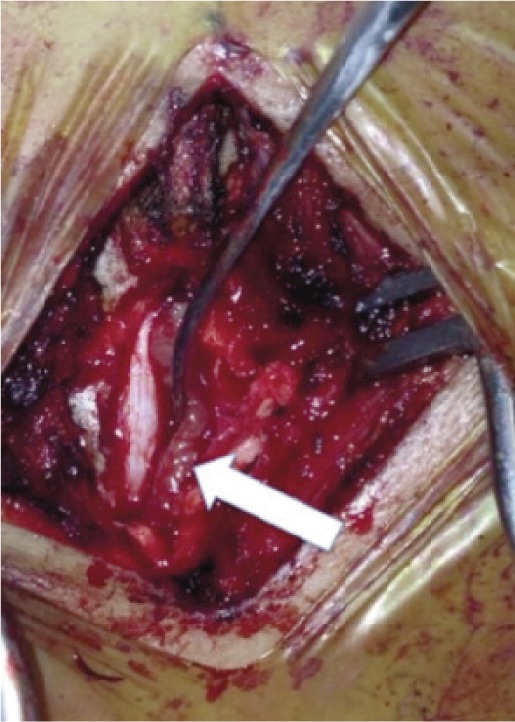
Intraoperative picture after laminectomy of T10 and T11. Arrow showing the partially elevated soft tissue encasing the dura.

Successful clearance of the soft tissue exposed an intact, healthy, shiny and pulsating dura (Figure 4b). Bone fragments and epidural soft tissue were sent for histopathological examination, culture and sensitivity. A body cast was applied postoperatively.

**Fig. 4b:**
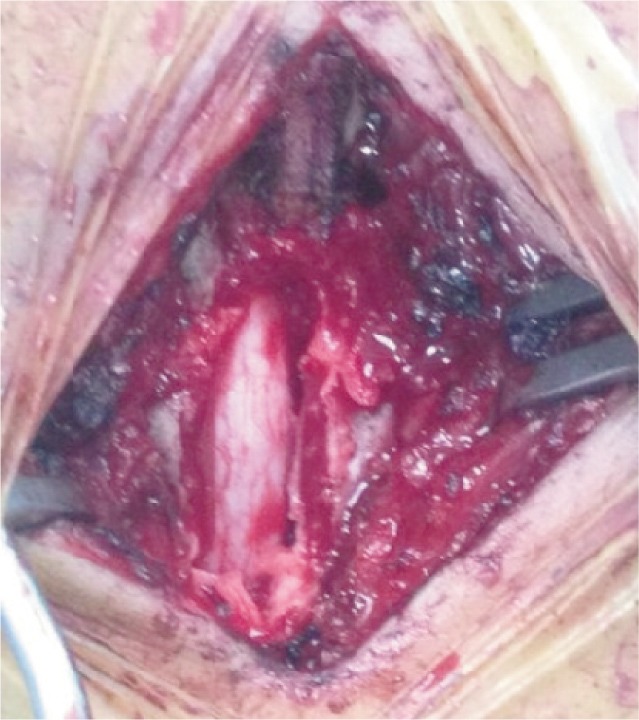
Intraoperative photograph showing the dura after soft tissue removal.

HPE was reported as caseating granulomatous inflammation (Figure 5a, 5b). No organisms were cultured. Ziehl-Neelson staining was negative. It was concluded that this child had extradural tuberculoma with neurological manifestation without any spinal deformity and relatively normal radiographs.

**Fig. 5a:**
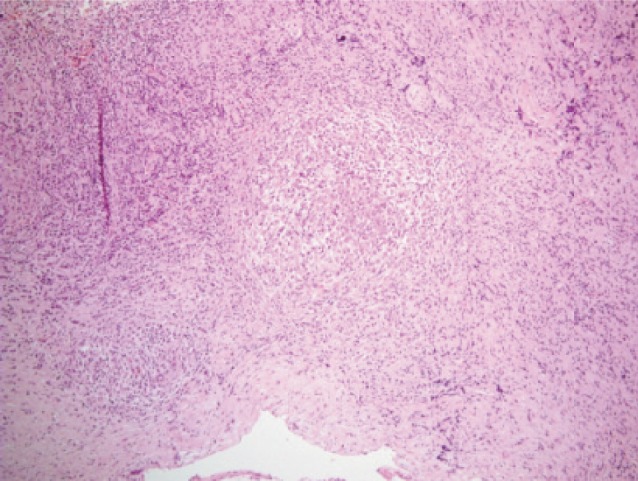
HPE showing granulomas.

**Fig. 5b:**
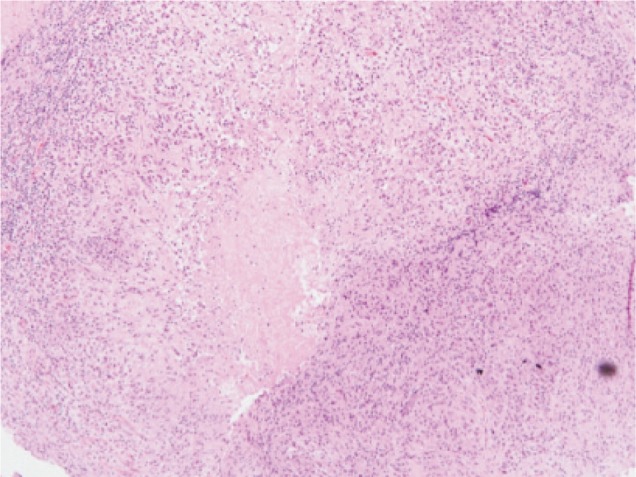
HPE caseous necrosis.

Postoperatively, anti-TB chemotherapy was initiated. On the 2nd post-operative day, her motor power at L2 myotome improved by two grades; however L3 and below were still grade 0. Her rehabilitation was continued. On the 12th week post-operative follow-up, the body cast was removed. She was able to ambulate independently with ataxic gait. Neurological assessment revealed MRC grade 4 power for L2 to L4, grade 3 for L5 and S1 bilaterally. Sensation was normal for from L1 to L3, reduced for L4 and L5 and absent for S1.

At 25th week post-operative follow-up, neurological assessment revealed MRC grade 5 power and intact sensation from L2 to S1 bilaterally. She was able to ambulate independently with normal gait.

Although she has now been successfully treated with full neurological recovery, she is still under our follow-up to monitor her local spinal vertebral growth which may have been by the inflammatory process. Currently her spinal alignment is still maintained and there is no evidence of late onset deformity at 2 years follow-up.

## Discussion

A toddler with a healthy family and minimal contact with the community outside her home would be considered to have very low risk of contracting tuberculosis. Her initial presentation of low grade fever and non-specific abdominal and back pain is not an uncommon presentation in the paediatric age group. This resulted in delay of initiation of special investigations until she progressed to paraplegia seven weeks from her initial symptom. Nevertheless, it would be unrealistic for all paediatric patients with low grade fever and back pain to undergo MRI. In view of these facts, spinal tuberculosis of the spine in the paediatric age group usually presents late or after the onset of neurology. Spinal tuberculosis can be treated with chemotherapy with good outcome as published in the 14th report of the Medical Research Council Working Party on Tuberculosis, 1999.

A high index of suspicion could prompt an earlier MRI request. The typical MRI features for TB spine are well defined paraspinal abnormal signals, a thin and smooth abscess wall, presence of paraspinal and intraosseous abscess, subligamentous spread of three or more vertebral levels, thoracic vertebral body involvement and hyperintense on T2 weighted images^2^. Our patient’s MRI findings differs as she had thick walled abscess. Other features were similar.

Tissue diagnosis is not absolutely compulsory for spinal tuberculosis. Spine tuberculosis can be diagnosed clinically, supported by imaging^3^. However, positive tissue culture would further fortify the diagnosis and above all, provide us with the extremely important chemotherapy sensitivity, especially with the emergence of multi drug resistance tuberculosis (MDR-TB). WHO reported the median prevalence of primary and acquired MDR-pulmonary TB to be 3.4% and 25%.

Spinal tuberculosis is a severe form of extrapulmonary tuberculosis, a Category I on the WHO classification. Treatment of Category I is two months of isoniazid, rifampicin, pyrazinamide and ethambutol, followed by four months of isoniazid and rifampicin. The tubercle bacilli isolated show four different types of growth kinetics and metabolic characteristics, leading to utilization of multiple drugs chemotherapy regime^4^.

For ease of decision making and management, spinal tuberculosis can be broadly classified into two groups of lesions: those with neurological complications and those without. In patients without neurological deficits, medical therapy is the treatment of choice and surgical intervention may be needed in relatively few cases. In cases with neurological complications, medical therapy is the first choice again but when indicated, combination of medical and surgical treatments yield the best results. Laminectomy is recommended in patients with posterior complex disease and spinal tumor syndrome. Late onset paraplegia is best prevented by early diagnosis and appropriate treatments^1^.

Surgical procedure decided for this patient was posterior decompression alone as she presented with extradural tuberculoma with no features of instability or deformity^5^. Spinal cord oedema and T10-T11 epidural abscess produced the clinical features of neurological deficits below T11 level. There were disc destruction, end plate erosions and marrow changes in the MRI. However spinal alignment was maintained and there were no features of structural instability. In view of the absence of preexisting radiographic spinal instability features, we were confident that a 2-level thoracic laminectomy decompression with facet preservation procedure would not cause spinal instability. We do acknowledge spinal infections and malignancies in a stable spine, unless well managed, would eventually lead to instability. Nevertheless, a body cast was applied for pain relief and also to maintain a good spinal alignment especially in this young patient with doubtful instruction compliancy.

In conclusion, a high index of suspicion for spinal tuberculosis, especially in endemic countries, could hasten the diagnosis and shorten the delay in initiating appropriate treatment. The mainstay of spinal tuberculosis treatment is still chemotherapy^1^. The many surgical options available should be considered on case to case basis and should be carefully planned to avoid under or over treatment. Surgical options ranges from a stand-alone posterior decompression to radical anterior-posterior debridement and reconstruction. Long term follow-up is necessary for early detection and timely intervention to prevent late onset deformity and dreadful irreversible neurological complications.
